# Nondestructive residual stress depth profile analysis at the inner surface of small boreholes using energy-dispersive diffraction under laboratory conditions

**DOI:** 10.1107/S1600576720014508

**Published:** 2021-02-01

**Authors:** Christoph Genzel, Matthias Meixner, Daniel Apel, Mirko Boin, Manuela Klaus

**Affiliations:** a Helmholtz-Zentrum Berlin für Materialien und Energie, Germany

**Keywords:** residual stress, energy-dispersive diffraction, inner surface of boreholes, nondestructive investigation, depth profile analysis

## Abstract

Energy-dispersive diffraction under both laboratory and synchrotron conditions was applied to study the hoop stress in the near-surface region of the inner wall of boreholes with a small diameter of 2 mm. By use of different X-ray beam cross sections for the sin^2^ψ measurements, it is demonstrated that the borehole-to-beam-diameter ratio must be considered in the evaluation. A beam cross section which is comparable to the borehole diameter reduces the slope of the *d* *^hkl^*_φψ_–sin^2^ψ distributions and thus invalidates the result of stress analysis. A quantitative relationship is applied, which allows the results obtained under the above conditions to be scaled so that they reflect the actual residual stress state at the measurement position. Owing to the small diffraction angles, energy-dispersive diffraction proves to be the only suitable experimental technique that allows a nondestructive and depth-resolved analysis of the hoop stress component at the inner surface of boreholes with a large length-to-diameter ratio.

## Abstract 2

Energy-dispersive diffraction is used for the nondestructive analysis of the hoop stress depth distribution at the inner surface of narrow boreholes. The results are corrected for the rotation effect, which is shown to have a significant influence on the measurements.

## 1. Introduction   

Residual stress analysis of polycrystalline materials by means of diffraction methods has been established as a powerful tool for many decades. Depending on the probe used for the measurements, diffraction methods enable the nondestructive and phase-selective evaluation of the residual stress state in different material zones (Noyan & Cohen, 1987 ▸; Hauk, 1997 ▸; Fitzpatrick & Lodini, 2003 ▸). While the sample surface plays a rather minor role in the case of neutron diffraction applied to stress analysis in the bulk, it can significantly affect the results of X-ray stress analysis (XSA) performed in reflection geometry, where the information depth is limited to the surface region. Instrumental parameters such as the equatorial and axial divergence of the primary beam result in a shift of the diffraction lines [see *e.g.* Alexander (1948 ▸, 1950 ▸), Eastabrook (1952 ▸) and Wilson (1965 ▸)], which may lead to considerable ‘ghost stresses’ if XSA is performed using the 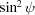 method (Macherauch & Müller, 1961 ▸) in the asymmetric Ω geometry (Zantopulos & Jatczak, 1970 ▸; Faninger, 1976 ▸). The influence of geometrical sources of error such as wrong sample height and/or beam position for both Ω and Ψ geometries has been investigated (Fenn & Jones, 1988 ▸; Jo & Hendricks, 1991 ▸; Convert & Miege, 1992 ▸), and pronounced surface profiles generated, for example, by turning may also affect the results of XSA experiments (Doig & Flewitt, 1981 ▸).

While the focus of the above-mentioned work is on the consideration of plane samples, the situation becomes even more complicated if the surface exhibits a pronounced convex, concave or toric curvature. This is often the case for technical components with complex geometry such as wires, springs, or parts with notches or grooves. In these cases, the results of XSA measurements are influenced by four effects, which depend on the respective sample geometry and whose influence may vary depending on the specific boundary conditions. These effects arise from (*a*) the rotation of the local reference system, (*b*) its translation from the diffractometer center, (*c*) absorption, and (*d*) partial shadowing and/or screening of the X-ray beam (François *et al.*, 1992 ▸).

Much theoretical and experimental work has been done to investigate the influence of the above-mentioned effects. Many authors examine only some of these effects, such as the translation and/or absorption effect (Doig & Flewitt, 1978*a* ▸,*b* ▸; Dowling *et al.*, 1988 ▸; Yu & Zhang, 1989 ▸; Berruti & Gola, 2003 ▸; Rivero & Ruud, 2008 ▸) or the rotation effect (Willemse & Naughton, 1985 ▸). A holistic theoretical approach, which includes translation and rotation effects as well as absorption and shadowing, was formulated by François *et al.* (1995 ▸) and Dionnet *et al.* (1996 ▸, 1999 ▸). These authors developed a formalism which can be applied to XSA on bulk samples or thin layers featuring both concave and convex curvature. For the rotation effect, which takes into account the variation of the orientation of the local sample reference system, it is shown that the generalized X-ray elastic constants or stress factors, *F*_*ij*_, have to be modified.

Since even today most XSA measurements are performed in the angle-dispersive (AD) diffraction mode in reflection geometry using monochromatic radiation, this also applies to the investigations at curved surfaces reported in the literature. For several reasons in many cases large diffraction angles 2θ are used, which enable measurements in back-scattering geometry. In this way, the irradiated area on the sample surface can be kept small and beam shadowing can often be avoided. Furthermore, the lattice strains to be analyzed lead to large and easily detectable shifts Δ2θ of the diffraction lines. As several of the publications cited above show, such measurement configurations are well suited for the determination of the residual stress component on curved surfaces in both tangential (circumferential) and axial directions. However, the precondition is free access to the measurement point, *i.e.* the surface to be analyzed must not be hidden, as is the case, for example, for the inner surface of tubes or boreholes.

In these cases the incident and diffracted beams must pass through the tube or borehole. For a large length-to-diameter ratio, this implies that the measurements have to be performed at small diffraction angles, which in practice excludes the AD diffraction mode in most cases. Moreover, owing to shadowing effects the analysis is usually restricted to the hoop stress component acting in the tangential direction. Under such limited boundary conditions only the energy-dispersive (ED) diffraction method (Giessen & Gordon, 1968 ▸; Buras *et al.*, 1968 ▸) is suitable for the analysis of the residual stress state. Its decisive advantages compared with the AD method are that complete diffraction spectra are determined under fixed, but nevertheless freely selectable, diffraction angles, which are usually small owing to the high photon energies and lie in a range between about 6 and 20° (Genzel & Klaus, 2017 ▸). Since the individual reflections *hkl* in the diffracted spectrum originate from different average depths below the surface, the ED method allows for a nondestructive analysis of the near-surface residual stress state, if the 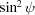 method is applied to each diffraction line in the spectrum (Genzel *et al.*, 2004 ▸).

With this paper we address the following issues. Starting with a formulation of the residual stress state at a curved surface in cylindrical coordinates (Section 2.1), we define the ED diffraction geometry boundary conditions under which 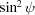 measurements can be performed on the inner surface of boreholes with large length-to-diameter ratio (Section 2.2). Then, the influence of the translation and rotation effects is discussed for ED-XSA (Section 2.3). Whereas the former effect is of minor importance in ED diffraction and can be controlled by calibration using stress-free powder applied to the curved surface, the latter considerably affects the result of X-ray stress analysis. This question is addressed in Section 2.4 by proposing a modification of the fundamental XSA equation which can be applied if certain assumptions about the residual stress state within the irradiated surface region are fulfilled. Using measurements performed under both laboratory and synchrotron conditions (see Section 3) on a small borehole, we demonstrate in Section 4 that ED diffraction allows the nondestructive acquisition of residual stress depth profiles of its inner surface even under laboratory conditions if the results of the 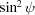 analysis are scaled with a factor that is determined by the ratio of the X-ray beam cross section to the borehole diameter. The paper closes in Section 5 with some conclusions from the present investigations.

## 2. X-ray residual stress analysis at the inner surface of boreholes   

### 2.1. Residual stress state at a cylindrical surface   

**Figure 1:**
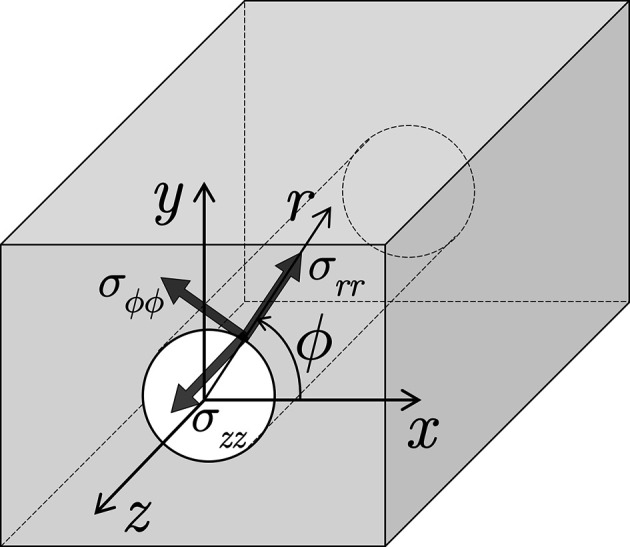
Description of the residual stress state at the inner surface of a borehole in cylindrical coordinates. Shown are only the normal stress components in the circumferential, radial and axial directions, σ_ϕϕ_, σ_*rr*_ and σ_*zz*_, respectively.

We consider the inner surface of the borehole shown in Fig. 1 ▸. The rotational symmetry with respect to the center axis of the borehole suggests a description of the stress/strain state in cylindrical coordinates (*r*, ϕ, *z*) (Gil-Negrete & Sanchez-Beitia, 1989 ▸). The stress equilibrium equations then read (Timoshenko & Goodier, 1951 ▸)
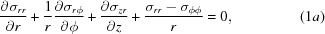


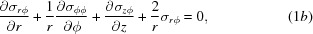


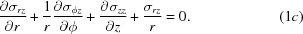
In the following the residual stress state at the inner surface is assumed to be of rotational symmetry and homogeneous with respect to the axial *z* direction, *i.e.* ∂/∂ϕ = ∂/∂*z* = 0. If it can be further assumed that no shear stress components occur in the irradiated sample volume, *i.e.* σ_*r*ϕ_ = σ_*z*ϕ_ = σ_*zr*_ = 0, then the above equilibrium conditions are reduced to the following expression:
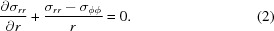
The above equation indicates that the near-surface residual stress state for specimens with cylindrical shape must be considered multi-axial, because of the term connecting the radial and the hoop stress components.

With the assumptions on the residual stress state made above (no shear components), the fundamental equation of XSA for a 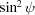 measurement in the circumferential direction takes the form
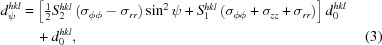
where 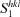 and 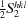 are the diffraction elastic constants. It should be emphasized that the stress component σ_*rr*_ cannot be neglected *a priori* in the evaluation via equation (2), as is often done for the σ_33_ component in XSA measurements at flat surfaces. However, since σ_*rr*_ must be zero directly at the surface, it can only occur as a gradient, the steepness of which depends on various parameters such as the manufacturing process, the material’s microstructure and the surface treatment. Therefore, the occurrence of the σ_*rr*_ component within the rather small information depth accessible by means of X-ray diffraction must be considered separately for each specific case. Since we found no evidence for the occurrence of a radial stress component in our experimental investigations (Section 3), we will confine our considerations in the following to a biaxial stress state, *i.e.* the stress component σ_*rr*_ will be omitted in the further equations.

### 2.2. Geometrical constraints   

**Figure 2:**
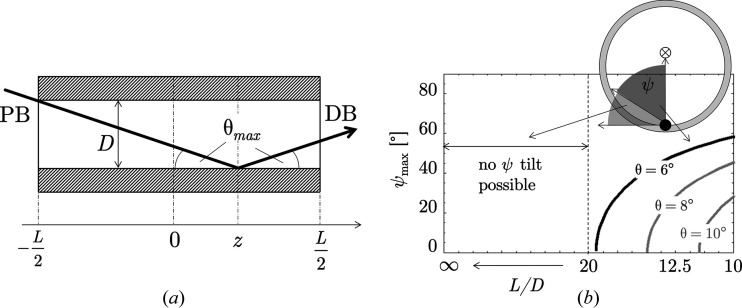
Schematic representation of the geometric conditions for X-ray residual stress analysis on the inner surfaces of tubes and boreholes. (*a*) Longitudinal section with X-ray beam path (PB, DB – primary and diffracted beams; *D*,* L *– diameter and length of the borehole). (*b*) View from the direction of the incident beam.

Inner surfaces of boreholes featuring a large length-to-diameter ratio are a considerable challenge for XSA measurements. Fig. 2 ▸ illustrates the geometrical situation for a 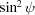 measurement at some point *z* on the inner surface of a tube of length *L* and diameter *D*. Assuming symmetrical diffraction conditions, it can be seen from Fig. 2 ▸(*a*) ▸ that the maximum Bragg angle for which both the primary and the diffracted beam can pass through the tube without shadowing depends on the measuring position *z*:
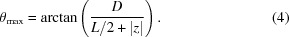
However, to ensure a shadow-free sample tilt up to sufficiently large inclination angle ψ, the Bragg angle used for residual stress analysis must be smaller than θ_max_. Obviously, the maximum tilt angle ψ_max_ becomes a function of θ, *z* and the length-to-diameter ratio 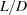: 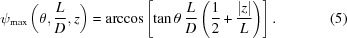
The above equation and the illustration in Fig. 2 ▸(*b*) show that the shorter the tube segment or borehole to be investigated, the more favorable the conditions for the residual stress analysis become.

It is evident from the above considerations that ED diffraction is the only method that provides the appropriate features for XSA measurements under these boundary conditions. Bragg’s law in its energy-dispersive form reads (Giessen & Gordon, 1968 ▸)
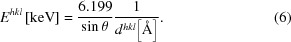
It relates the lattice spacing *d* ^*hkl*^ to be evaluated to the energy position *E* ^*hkl*^ in the diffraction pattern measured for a fixed Bragg angle θ. For analyses on body-centered cubic ferritic steel (strain-free lattice parameter *a*_0_ = 2.8665 Å) at an angle θ = 8°, the strongest interference lines are in an energy range between 22 keV (110) and 58 keV (321) and can therefore be measured with the *Bremsstrahlung* spectrum of a conventional tungsten X-ray tube. Furthermore, assuming a ratio 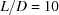, 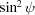 measurements can be performed up to a tilt angle ψ = 45°, which is sufficient for a depth-resolved residual stress analysis using the modified multi-wavelength method (Klaus & Genzel, 2019 ▸). Note that under these geometrical conditions only the hoop stress component is accessible by means of a 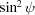-based analysis.

### 2.3. Influence of the translation and rotation effects: qualitative discussion   

The impacts of both effects have been investigated in detail for the case of AD diffraction in various publications either individually or together (see *Introduction* [Sec sec1]). Concerning the translation effect, which is caused by the deviations of parts of the scattering volume from the goniometer center, ED diffraction provides some advantages compared with the AD mode. This is because the diffracted spectrum is recorded under a fixed angle 2θ. Consequently, the equatorial divergence of the diffracted beam can be confined by slit systems to very small values <0.01°. Thus, shifts Δθ of the Bragg angle in equation (6) due to the translation effect are negligible in practice. However, ED-XSA performed under laboratory conditions featuring a reduced photon flux compared with synchrotron radiation requires larger beam cross sections. This leads to an increase of the divergence and, thus, calibration measurements on stress-free powder applied to the curved surface have to be carried out to eliminate the translation effect.

**Figure 3:**
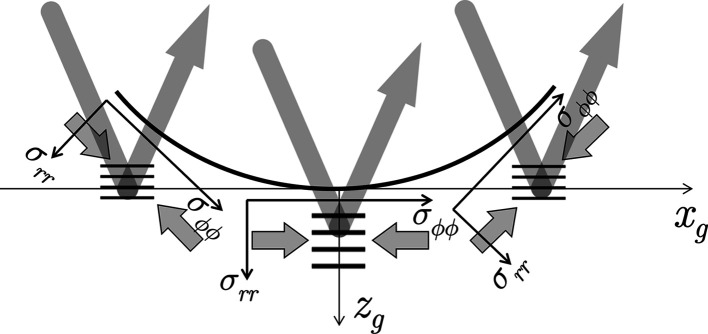
X-ray residual stress analysis (case ψ = 0 with respect to the global sample reference system denoted by the subscript ‘g’ on the coordinate axes) at a curved sample surface. σ_ϕϕ_ and σ_*rr*_ are the in-plane (hoop) and out-of-plane (radial) normal stress components, respectively, in the different local reference systems. The angled arrows mark the pathways of the primary and the diffracted beams.

In contrast to the translation effect, the rotation effect influences AD-XSA and ED-XSA measurements in the same way. The situation is shown in Fig. 3 ▸. The curvature of the surface leads to a local rotation of the principal axis system of the stress tensor relative to the global sample reference system. Therefore, if the near-surface sample area irradiated by the X-ray beam is located in a region of strong curvature, the lattice strains determined from the position of the diffraction lines always represent average values over different orientations, which can also be interpreted formally as different inclination angles ψ.

**Figure 4:**
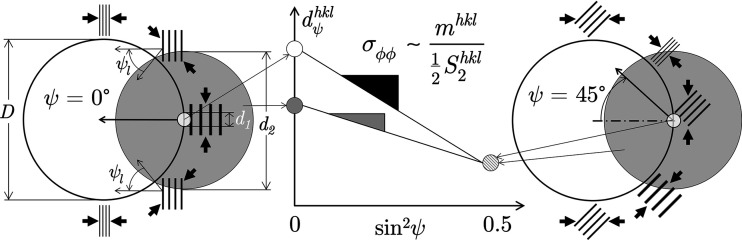
Schematic view showing the influence of a curved sample surface on the analysis of the hoop stress component by means of a 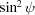 measurement. *D* – inner diameter of the tube or borehole; *d*_1_, *d*_2_ – small and large primary beam cross sections, respectively. The hoop stress is assumed as compressive and homogeneous along the circumferential direction.

The influence of a strongly curved surface on the 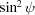 analysis will be explained by means of Fig. 4 ▸. The stress state is assumed to be uniform in the local reference systems in which the hoop and the radial stress components are defined. We now consider two different scenarios. In the first scenario the primary beam cross section *d*_1_ is much smaller than the hole diameter *D*. The illuminated area on the inner surface, especially the part in the circumferential direction, is supposed to be small. Performing a 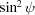 measurement under these conditions would result in a 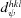
–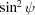 regression line whose slope is proportional to the actual residual stress state at the measuring point.

In the second scenario the primary beam cross section *d*_2_ is comparable to the hole diameter *D*. Now, the irradiated part of the surface along the circumferential direction becomes larger and the lattice planes that fulfill the Bragg diffraction condition for each inclination angle ψ have different orientations with respect to the (curved) surface. This means, however, that a distinction must now be made between a ‘global’ ψ angle (*i.e.* the value set for the measurement) and ‘local’ ψ_l_ angles, which vary continuously with the surface curvature. Consequently, the lattice spacing obtained from the position of the diffraction line according to equation (6) is an average over various orientations with respect to the local reference system of the stress tensor.

Comparing the left and right hand side drawings of Fig. 4 ▸, it becomes clear that the averaging has different consequences for ψ = 0 and ψ ≠ 0. In the first (‘symmetric’) case, the X-ray beam captures smaller lattice spacings 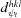 on both sides of the central region. Thus, the corresponding mean value 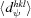 is smaller than the central value 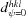. For ψ ≠ 0 (‘asymmetric case’) the lattice spacings 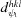 captured on either side of the central area are larger and smaller, respectively, than the central value 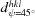. Hence, the average lattice spacing 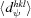 should be comparable to the central value. In summary, as can be seen from the schematic 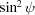 plot in Fig. 4 ▸, a smaller slope of the regression line is to be expected if the measurement is performed using a large beam cross section.

### 2.4. Impact of the rotation effect on residual stress evaluation   

#### 2.4.1. Modification of the fundamental equation of X-ray stress analysis   

**Figure 5:**
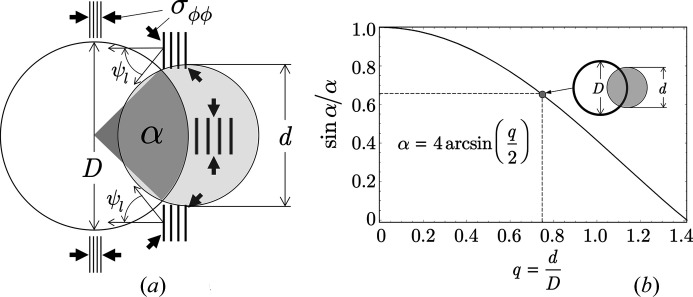
Illustration of XSA on strongly curved surfaces. (*a*) The angle α marks the range of ‘local’ orientations ψ_l_ that are captured by the primary beam for some ‘global’ tilt angle ψ adjusted with the diffractometer setup (here ψ = 0). σ_ϕϕ_ denotes the hoop stress component to be analyzed. (*b*) Correlation between the ratio *q* of the beam cross section *d* to the hole diameter *D* and the scaling factor 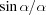 (α to be taken in radians).

For the evaluation of a 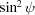 measurement performed at the inner surface of a borehole using an X-ray beam with small cross section (*d*_1_ in Fig. 4 ▸) the fundamental equation of XSA given by equation (3) has to be applied. For this configuration the analysis would provide the actual value for the hoop stress component, σ_ϕϕ_, since the local and global reference systems are coincident. However, if the measurement is carried out using a large beam cross section comparable to the hole diameter (*d*_2_ in Fig. 4 ▸), the irradiated part of the inner surface can be described by an angle α [Fig. 5 ▸(*a*) ▸]. In order to capture all lattice spacings that simultaneously fulfill the Bragg condition but are assigned to different ‘local’ angles ψ_l_, it is necessary to integrate over all orientations 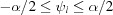: 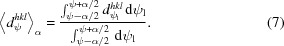
If in the above equation for 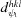 the right side of equation (3) is inserted (note: the radial stress component σ_*rr*_ will be omitted), the following relation is obtained:
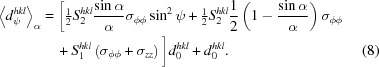
Equation (8) remains linear in 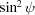 but the slope and the intercept with the ordinate axis now depend on a scaling factor 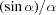. Because 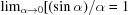, equation (8) takes the usual form (3) for very small beam cross sections.

The above equation requires a thorough discussion. It represents a special case of the general solution for the rotation effect developed by François *et al.* (1995 ▸) and Dionnet *et al.* (1999 ▸). Dionnet *et al.* (1999 ▸) also consider two special scenarios, which refer to different absorption models. Both models are based on two assumptions: (1) The irradiated surface remains constant during the 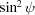 measurement performed in the symmetrical Ψ mode; this can be fulfilled, for example, by the use of absorbing masks which confine the part of the surface to be investigated (Oguri *et al.*, 2000 ▸, 2002 ▸). (2) While taking absorption into account generally requires integration over the irradiated sample volume according to Beer’s law, the treatment can be reduced to surface integrals if the penetration depth of the X-rays is small compared with the sample radius (‘thick specimen approximation’), or if the thickness of the examined material is small compared with the penetration depth of the X-rays (‘thin specimen approximation’) (François *et al.*, 1995 ▸). The boundary condition 1 (irradiated surface remains constant) implies that the intensity decrease during a 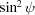 measurement in the thick specimen approximation has to be considered in the evaluation in the form of a weighting factor, while the intensity in the thin specimen approximation remains constant.

For the case considered in this paper the situation is reversed, because the irradiated inner surface cannot be confined for geometrical reasons to a constant value (long borehole with small diameter) but changes during the 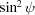 measurement according to 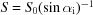. *S*_0_ and α_i_ are the primary X-ray beam cross section and the incidence angle between the surface and the X-ray beam, respectively. For the symmetrical Ψ mode one finds 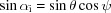. The total diffraction power *P* ^D^ of a homogeneous sample or film of thickness *D* then becomes (Klaus & Genzel, 2013 ▸)
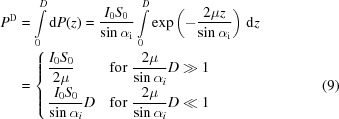
where *I*_0_ and μ are the primary beam intensity and the linear absorption coefficient, respectively. From the above equation it can be seen that the total diffraction power does not depend on the incidence angle for the thick specimen approximation [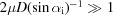], but it increases for the thin specimen approximation [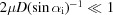] with decreasing α, which is due to the enlargement of the irradiated volume of the thin layer. Therefore, the case considered in equation (8) corresponds to the ‘*I*′ = constant’ case for the hoop stress component described by equation (12) of Dionnet *et al.* (1999 ▸).

The influence of the scaling factor will be illustrated by a numerical example. The following scenario is based on real conditions, as demonstrated in Section 4.1 by means of experimental examples. We consider a ferritic steel sample with a 2 mm borehole featuring a uniform biaxial residual stress state of −1000 MPa in the near-surface region of the inner surface, which could be generated, for example, by mechanical surface treatment such as shot-peening. Let us further suppose that the white X-ray beam used for the investigation has a cross section of 1.5 mm. The ratio 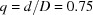 thus corresponds exactly to the situation shown in Fig. 5 ▸. It corresponds to an angle 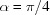 and, thus, to a scaling factor 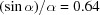. This means, however, that the slope of the 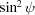 regression line would be reduced by this factor and the analysis for the hoop stress component σ_ϕϕ_ would only result in a value of −640 MPa.

#### 2.4.2. Special cases   

**Figure 6:**
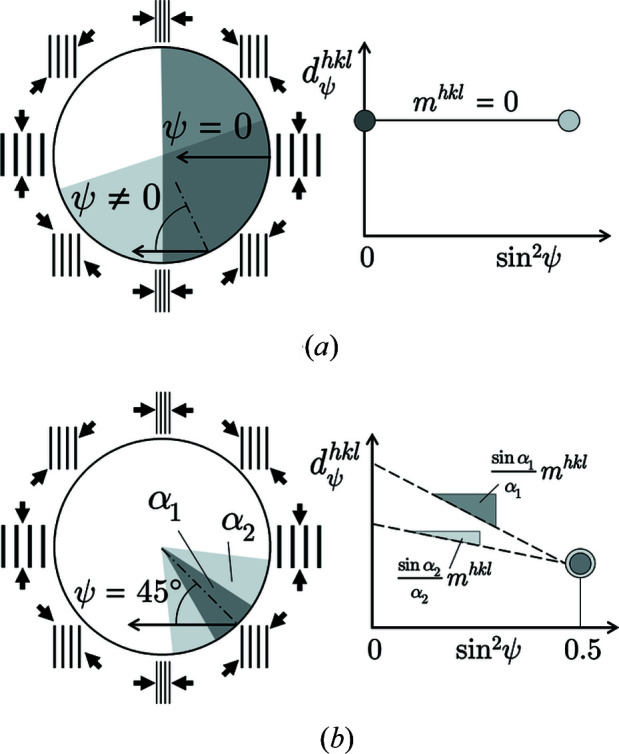
Schematic view of two scenarios that follow directly from equation (8). (*a*) Hypothetical limiting case α = π and ψ variable. (*b*) Fixed inclination angle ψ = 45° and variable α.

In the previous section it was shown that the result of a 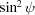 analysis performed at the inner surface of a borehole obviously depends on the size of the beam cross section used for the experiment. In this section, we will show what consequences result from equation (8) under certain conditions. The two cases shown in Fig. 6 ▸ result from setting α = π (case a) and ψ = 45° (case b), where the other variable can be freely selected. Both cases lead to the same result:
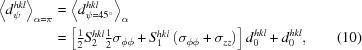
which, however, must be interpreted differently depending on the assumption made in each case. Case (a) is the hypothetical limit case, according to which averaging the lattice strains over half the circumference of the hole (assuming a homogeneous stress state) always yields the same value, regardless of the ψ angle selected. The slope of the 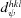
–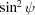 distribution is therefore zero. From a practical point of view, case (b) is more interesting. If measurements are made under ψ = 45°, the homogeneity of the stress state along the inner surface can be checked by varying the angle α (adjustable via the beam cross section).

## 3. Experimental   

In order to verify the theoretical considerations in the previous sections, experimental investigations were carried out on boreholes made in ferritic steel with defined residual stress state under various conditions with regard to the primary beam cross section. Because the sample material in the present case serves only as a ‘means to an end’ and comes from an industrial series production, the manufacturing conditions and the intended use of the investigated components will not be discussed further, since this information is not relevant for answering the questions of interest here.

### 3.1. X-ray diffraction setup   

#### 3.1.1. Laboratory   

**Figure 7:**
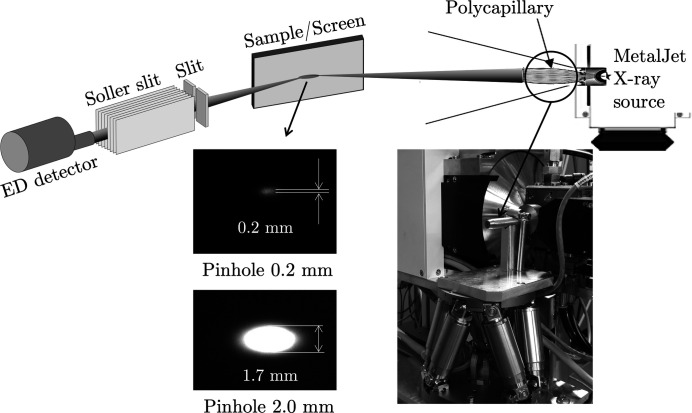
Top: schematic view of the diffraction geometry using the MetalJet X-ray source for the XSA experiments. Bottom left: size of the primary beam at the measurement location, taken with a fluorescent screen set to 24° (*i.e.* the vertical extension in the image corresponds to the true beam cross section). Bottom right: photograph of the MetalJet source. The arrow points to the polycapillary, which is adjusted by the hexapod below. Note that the actual beam cross section at the measuring point is slightly smaller than the diameter of the pinhole used to confine the primary beam at the exit side of the polycapillary, which is due to the focusing effect of the lens.

Most of the measurements were performed under laboratory conditions exploiting the white *Bremsstrahlung* spectrum emitted by a high-flux MetalJet X-ray source developed by the company Excillum. Table 1 ▸ summarizes the important parameters. The liquid metal jet, which serves as the anode, is a mixture that mainly consists of gallium (∼80%) and indium (∼20%). The geometrical beam path, the horizontal diffraction geometry and the arrangement of the optical elements can be seen in Fig. 7 ▸. The large focal length *f*_2_ on the exit side of the polycapillary lens and the resulting large distance of the source from the sample serve to keep the divergence in the primary beam as small as possible in order to prevent geometrically induced line broadening (Genzel & Klaus, 2017 ▸).

Sample positioning during the 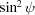 measurements was realized by means of a three-circle diffractometer consisting of a large ω-rotation table on which a closed Eulerian cradle with integrated ϕ-rotation and *x-y-z*-translation tables is mounted. A laser and CCD camera system is available for sample adjustment. However, since the measuring point on the inner surface of the boreholes was not visible from the outside, the alignment in the present case was carried out using a through-surface scanning procedure (see Section 3.1.3). The detector is mounted on an *x-y-z*-translation stage which also allows for rotation in the horizontal diffraction plane to adjust the diffraction angle 2θ.

#### 3.1.2. Synchrotron   

A drawback of ED diffraction experiments performed under laboratory conditions using the white *Bremsstrahlung* spectrum emitted by a solid or even by a liquid anode is the lower photon flux compared with synchrotron X-rays. Owing to the very high photon flux, sufficient counting statistics can be achieved even for very small beam cross sections when using a synchrotron. For gauging the impact of the spot size on the residual stress analysis on strongly curved surfaces, this means that synchrotron measurements can serve as a reliable reference.

We are therefore fortunate that, before closure of the energy-dispersive materials science beamline EDDI@BESSY II (Genzel *et al.*, 2007 ▸) in mid-2018, we still had the opportunity to perform XSA measurements on the component structures presented here, which can now be compared with the laboratory measurements. In contrast to the laboratory experiments the corresponding synchrotron measurements were done in vertical diffraction geometry, because of the linear polarization of the synchrotron beam in the storage ring plane. The measurements were performed for 2θ = 16° in the symmetrical Ψ mode up to ψ = 71.5° using the detector specified in Table 1 ▸. The counting time was 200 s per spectrum. The primary beam was confined by slits to about 100 × 100 µm. The equatorial divergence in the diffracted beam was confined by a double-slit system with apertures of 30 µm (equatorial) and 8 mm (axial) to <0.01°.

Owing to the larger energy range provided by the 7 T multipole wiggler, additional diffraction lines with higher photon energies could be included in the evaluation compared with measurements under laboratory conditions. Table 2 ▸ summarizes the energy positions and the maximum information depths 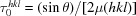 for the diffraction lines *hkl* that were taken into account for XSA under laboratory and synchrotron conditions (marked by a cross). Some diffraction lines could not be evaluated because of their weak intensity (222, 400, 420) or, as was the case for the 110 reflection, had to be excluded from the analysis because of overlap with other reflections.

#### 3.1.3. Sample alignment   

**Figure 8:**
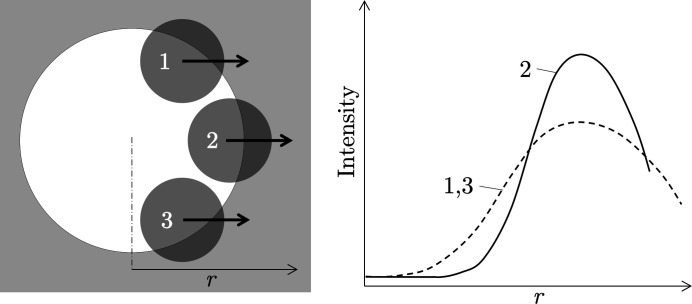
Schematic view of the sample alignment procedure. The white circle and the small gray circles mark the cross sections of the borehole and the X-ray beam, respectively. See text for details.

As already mentioned in Section 3.1.1 sample alignment for a stress measurement at the inner surface of a small borehole represents a challenge since no optical tools such as a laser or CCD camera system can be used. Fig. 8 ▸ shows the strategy applied to find the correct height (position 2) for the measurement point. By means of through-surface scanning with the gauge, which is defined by the optical elements in the primary and diffracted beam, intensity distributions are obtained whose slope depends on the vertical position of the gauge within the borehole. The optimum position is reached when the gauge is immersed vertically in the surface. In this case the intensity curve shows the steepest slope and the highest intensity at the maximum. The final control is then performed by comparing the intensities of diffraction patterns recorded at the optimal height position at inclination angles ψ = ±45°.

Note that Girard *et al.* (2000 ▸) used the configuration depicted in Fig. 8 ▸ in order to adjust different orientations ψ by means of a two-circle Ω diffractometer. If measurements in the positions 1 to 3 are performed using a horizontal diffraction setup in symmetrical reflection geometry, shadowing effects due to sample tilting are avoided. Assuming a uniform residual stress state along the circumference, the positions 1 and 3 then correspond to orientations ±ψ in the global reference system, whereas position 2 corresponds to ψ = 0. Owing to the large diffraction angles this procedure can only be applied to open structures with either a concave or a convex surface, if the measurements are performed in the AD diffraction mode. However, in the case of ED diffraction with rather small diffraction angles it represents an interesting alternative to the classical 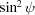 approach used in this paper.

## 4. Results   

### 4.1. Analysis under laboratory conditions   

**Figure 9:**
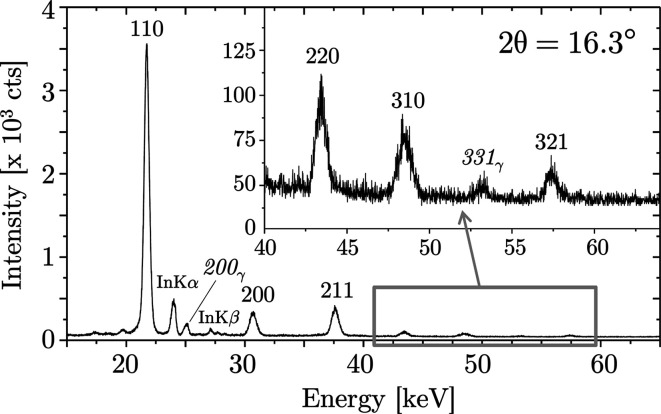
ED diffraction pattern of the investigated ferritic steel sample, measured under ψ = 0°. The indices written in italics are diffraction lines of the weakly represented retained austenite phase. The slight asymmetry of the 110 ferrite line is due to the superposition with the 111 interference of the retained austenite. It was therefore excluded from further evaluation.

The first example considered here refers to a borehole with a diameter of 2 mm and a length of 10 mm, the inner surface of which was mechanically treated by shot-peening. For the diffraction angle 2θ = 16.3° applied in the measurement, equation (5) yields the maximum tilt angle ψ_max_ ≃ 69°. However, under practical conditions, because of the extension of the X-ray beam, shadowing occurs earlier. Therefore, the maximum tilt angle range was confined to |ψ_max_| = 63°. The energy-dispersive diffraction pattern in Fig. 9 ▸ shows besides the diffraction lines originating from the sample also the characteristic X-ray lines of indium (*K*α = 24.2 keV, *K*β = 27.3 keV).

It is clearly recognizable from the depicted diffractogram that the focusing effect of the used polycapillary lens is limited to an energy range up to about 40 keV. In this range a high intensity of the diffraction lines is observed. For higher energies, the glass becomes transparent, resulting in increased absorption and thus a disproportionate weakening of the primary beam.

**Figure 10:**
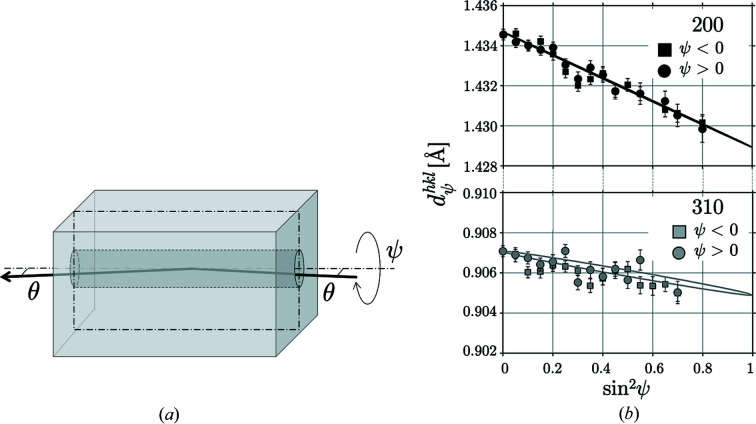
(*a*) Schematic view of the diffraction conditions for residual stress analysis on the inner surface of the borehole. The dash–dotted rectangle marks the cutting plane along which the sample was cut after completion of the nondestructive ED-XSA measurements. (*b*) Selected 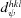
–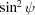 distributions.

Fig. 10 ▸ shows the geometrical arrangement used for the measurement and the 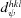
–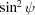 distributions for the reflections with the lowest (200) and the highest (310) photon energies considered in this example in the residual stress evaluation. The negative slopes of the regression lines reveal the occurrence of compressive residual stresses within the accessible depth range, which seem to decrease with increasing depth. ψ splitting, which would be an indication of the existence of shear stresses, is not observed.

**Figure 11:**
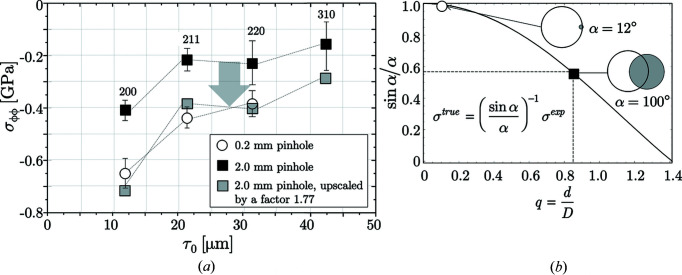
(*a*) Laplace stress depth profiles of the hoop stress component obtained under different conditions. (*b*) Borehole diameter to beam cross section ratios for the two 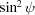 measurements. Note that the scaling factor 1.77 corresponds to 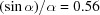, marked by the black square in (*b*).

The results of the XSA measurements on the borehole that were performed using different primary beam cross sections are summarized in Fig. 11 ▸. The diffraction elastic constants required in the evaluation were calculated from the single-crystal elastic con­stants for ferrite (Landoldt-Börnstein, 1984 ▸) by means of the Eshelby–Kröner model (Eshelby, 1957 ▸; Kröner, 1958 ▸). For intensity reasons the residual stress evaluation had to be restricted to three (0.2 pinhole) and four (2.0 pinhole) reflections, respectively. The discrete residual stress depth profiles 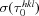 were obtained by means of the ‘multi-wavelength method’ (Eigenmann *et al.*, 1990 ▸), which has been modified for the ED diffraction case by Genzel *et al.* (2004 ▸). The basic idea of the ‘modified multi-wavelength method’ is to evaluate the linear range of the 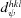
–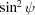 distributions according to the classical 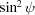 method and to plot the obtained stress values versus the maximum information depth 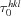 (*cf*. Table 2 ▸). In this way, a residual stress depth profile in the Laplace space is obtained (Klaus & Genzel, 2019 ▸), which is related to the actual depth of residual stress distribution in real space by the following relationship (Dölle & Hauk, 1979 ▸):
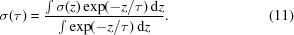
For the two measurement configurations considered here, the inverse of the scaling factor 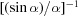 is 1.01 and 1.77 for the 0.2 and 2.0 mm pinhole, respectively [see Fig. 11 ▸(*b*) ▸]. Thus, the depth profile obtained for the small pinhole should reflect the actual residual stress state close to the inner surface of the borehole to a very good approximation and can therefore be used as a reference profile. From the diagram in Fig. 11 ▸(*a*) it can be seen that upscaling of the residual stress depth profile, which has been calculated from the measurements carried out with the large beam cross section, leads to a very good agreement with the reference profile.

### 4.2. Comparison with supplementary measurements   

**Figure 12:**
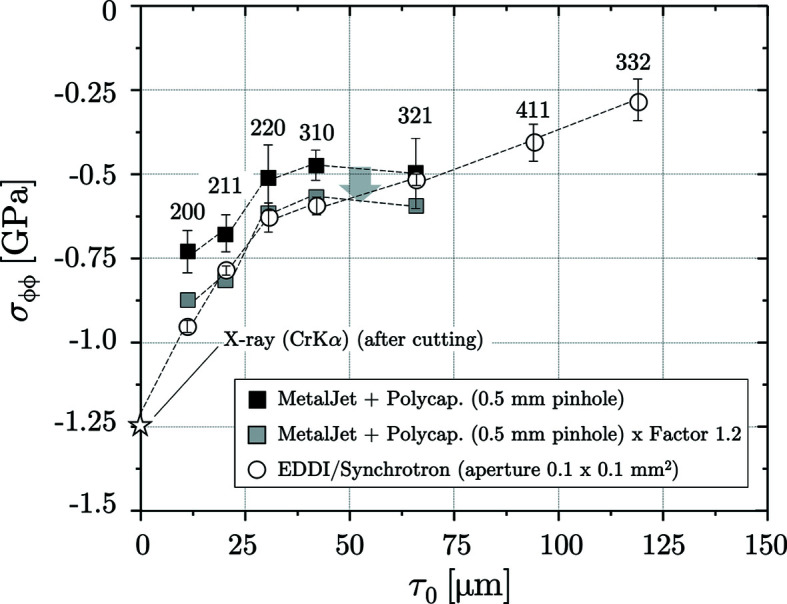
Comparison of X-ray stress analyses on a thin shot-peened borehole (same size and geometry as in the example shown in Figs. 9 ▸ to 11 ▸ ▸).

In Fig. 12 ▸ the measurements performed under laboratory conditions are compared with those obtained using synchrotron radiation. The results confirm the theoretical considerations regarding the relationship between the curvature radius of the inner surface of the borehole and the size of the beam cross section used for the measurement. Taking the synchrotron results as a reference and scaling the residual stress depth profile obtained in the laboratory by a factor of 1.2, which corresponds to the ratio of the borehole to the beam cross section diameter, provides a very good agreement.

After completion of the nondestructive ED-XSA measurements, the borehole was cut along its longitudinal axis [see Fig. 10 ▸(*a*) ▸] in order to analyze the hoop stress very close to the surface by AD-XSA using Cr *K*α radiation. Fig. 12 ▸ shows that the result of this measurement fits very well into the depth profile determined by ED-XSA. According to this, high compressive stresses generated by the surface treatment are present in the covered depth range in the circumferential direction, which reach values of about −1.25 GPa in the immediate surface region and decrease rapidly with increasing depth. The actual residual stress value at the surface before cutting may have been even higher, since cutting the investigated component into two halves may cause some relaxation of the macro residual stresses. However, in the present case it can be assumed that this relaxation is rather small, since the investigated component was very massive compared with the thin surface layer affected by residual stresses induced at the inner wall of the borehole by shot-peening. Therefore, an elastic spring-back associated with a rearrangement of residual stresses can almost be excluded.

## 5. Concluding remarks   

The aim of the investigations presented in this study was to show that energy-dispersive X-ray diffraction is the only suitable method for nondestructive and depth-resolved analysis of the residual stress state at the inner surface of narrow boreholes even under laboratory conditions. The sample alignment is very laborious and the analysis is based on a number of assumptions that must be fulfilled. Owing to the geometrical constraints the analysis is restricted to the hoop stress component. Since the irradiated surface cannot be limited by masks, it increases continuously during the 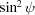 measurement. Therefore, it must be assumed that the residual stress state is uniform within the total area captured by the X-ray beam.

A further issue concerns the radial stress component, which cannot be neglected *a priori* since it is linked to the hoop stress component according to equation (2) in the case of surfaces featuring a strong curvature [see *e.g.* Atienza *et al.*( 2005 ▸)]. In the present case the near-surface residual stress state may be assumed to be approximately biaxial within the relatively small accessible depth range of about 100 µm. To prove this assumption, the depth of the lattice parameter in the strain-free direction ψ* of the biaxial stress state would have to be investigated (Genzel *et al.*, 2005 ▸). However, if the in-plane residual stress state does not have rotational symmetry (*i.e.* σ_ϕϕ_ ≠ σ_*zz*_), ψ* depends on the stress components themselves. Owing to the geometrical constraints the axial stress component σ_*zz*_ cannot be detected nondestructively at the inner surface of the borehole. Therefore, the result of the 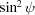 analysis for measurements of this kind is always the difference σ_ϕϕ_ − σ_*rr*_ between the hoop and radial stress components.

Under laboratory conditions the photon flux of the *Bremsstrahlung* spectrum that can be used for these experiments is low compared with that of a synchrotron. Therefore, sufficient counting statistics require beam cross sections comparable to the diameter of the boreholes. With the investigations presented here, it could be demonstrated that the rotation effect known from the literature under these conditions significantly influences the result of the 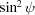-based stress analysis. The effect can be quantitatively described by a modification of the fundamental equation of XSA, which is based on simplifying assumptions regarding the absorption conditions. This has been verified by measurements using different X-ray beam cross sections. Applying the scaling factor calculated for the respective ratio of the X-ray beam to borehole diameter to the experimentally determined depth profiles, residual stress distributions are obtained for the individual measurements that are consistent within the error margins and measurement uncertainties.

## Figures and Tables

**Table 1:** Parameters used for the XSA experiments with the MetalJet X-ray source

Source	Liquid metal jet, 160 kV/1.56 mA (250 W), effective focus 20 × 20 µm
Optics (primary beam)	Polycapillary lens (*f*_2_ = 900 mm, δ = 0.26°); exit beam cross section defined by pinholes with diameters 0.2 and 2.0 mm
Optics (diffracted beam)	Equatorial Soller slit (δ = 0.15°) + 1.0 mm entrance slit
Detector	Ge semiconductor detector (Canberra model GL0110); resolution: 160 eV at 10 keV and 420 eV at 100 keV
XSA mode	Symmetrical Ψ mode (ψ = −63°…63°), Δ(sin^2^ψ) = 0.05
Diffraction angle	2θ = 16.3°
Integration time	300 s (∅ = 2 mm), 3600 s (∅ = 0.2 mm)
Calibration measurement	Stress-free W powder, applied at the measuring point and analyzed under identical conditions
Data evaluation	MATLAB-based software package *EDDIDAT* (Apel *et al.*, 2020 ▸)

**Table 2:** Energy positions and information depths 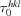 for ferritic steel for 2θ = 16°

*hkl*	*E* (keV)	τ_0_ (µm)	Laboratory	Synchrotron
110	22.0	4.5	–	–
200	31.1	12.0	×	×
211	38.1	21.5	×	×
220	43.9	32.3	×	×
310	49.1	43.9	×	×
222	53.8	56.1	–	–
321	58.1	68.9	×	×
400	62.1	81.9	–	–
411	65.9	94.7	–	×
420	69.9	107.6	–	–
332	72.9	120.7	–	×
